# Colonization of human opportunistic *Fusarium oxysporum* (HOFo) isolates in tomato and cucumber tissues assessed by a specific molecular marker

**DOI:** 10.1371/journal.pone.0234517

**Published:** 2020-06-12

**Authors:** Chao-Jen Wang, Chinnapan Thanarut, Pei-Lun Sun, Wen-Hsin Chung

**Affiliations:** 1 Department of Plant Pathology, National Chung Hsing University, Taichung, Taiwan; 2 Faculty of Agricultural Production, Division of Pomology Maejo University, Chiangmai, Thailand; 3 Department of Dermatology, Mackay Memorial Hospital, Taipei, Taiwan; Nanjing Agricultural University, CHINA

## Abstract

*Fusarium oxysporum* is a large complex cosmopolitan species composed of plant pathogens, human opportunistic pathogens, and nonpathogenic isolates. Many plant pathogenic strains are known based on host plant specificity and the large number of plant species attacked. *F*. *oxysporum* is an opportunistic pathogen in humans with a compromised immune system. The objectives of this study were: (1) to develop a specific marker to detect human opportunistic *F*. *oxysporum* (HOFo) isolates; (2) to determine whether or not HOFo isolates can colonize and cause disease symptoms in plants; and (3) to assess Taiwan isolates sensitivity to two agro-fungicides. The primer pair, Primer 5/ST33-R, specifically amplifying Taiwan and international reference HOFo isolates was developed and used to detect and assess the distribution of a Taiwan isolate in inoculated tomato plants and tomato and cucumber fruit. Taiwan HOFo isolate MCC2074 was shown to colonize tomato roots, hypocotyls, and cotyledons, but did not show any visible symptoms. Four days after surface inoculation of tomato and cucumber fruit with the same isolate, MCC2074 was detected in the pericarp and locular cavities of both tomato and cucumber fruit and in columella of tomato fruit. Three Taiwan HOFo isolates were found to be moderately sensitive to azoxystrobin and highly sensitive to difenconazole.

## Introduction

Filamentous fungal human pathogens (FFHPs), commonly found in clinics and hospitals [1], affect patients with suppressed immune systems, such as those with immune deficiency syndrome (AIDS), organ transplantation, and cancer chemotherapy [1, 2]. Among FFHPs, *Fusarium* spp. have been shown to cause fusariosis with disseminated infections in immunosuppressed patients resulting in mortality rates from 50 to 75% [3]. Twelve *Fusarium* spp. have been demonstrated to cause or link to human diseases, including keratitis, onychomycosis, dermatitis, and allergy [2]. Studies have showed that *F*. *solani* and *F*. *oxysporum* are two most dominant species accounting for 70% of infections and about 20% are associated with *F*. *verticillioidis* and *F*. *moniliforme* [2,4]. *F*. *solani* commonly causes human keratitis [5,6], while *F*. *oxysporum* is commonly found in onychomycosis [6–9]. Although anti-*Fusarium* spp. drugs are available, *Fusarium* spp. have recently been reported to develop resistance to various drugs in clinical cases [2,10]. Thus, therapeutic efficacy of clinical medicines on the suppression of *Fusarium* spp. is reduced [2,11]. Due to a wide distribution and the occurrence of drug-resistance of human opportunistic *Fusarium* spp., fusariosis becomes problematic and have raised a great deal of public awareness [3, 12–15].

*F*. *oxysporum* and *F*. *solani* are commonly present in soil, plant tissues and residues, organic medium, and water [16]. Strains of these two species are important plant pathogens that cause root rot, crown rot, stem rot, dry rot, yellowing, vascular discoloration and wilting in a wide range of plant species belonging to Gramineae, Solanaceae, Brassicaceae, and Cucurbitaceae [17–21]. *F*. *oxysporum* is one of the most important plant pathogens causing Fusarium wilt in crops worldwide [22,23]. A study has reported that *F*. *oxysporum* f. sp. *lycopersici*, the causal agent of fusarium wilt of tomato, causes disseminated infection and increases mortality rates in immunosuppressed mice [24]. Further studies indicated that *F*. *oxysporum* f. sp. *lycopersici* can colonize heart, kidney, lung, spleen, and liver in immunosuppressed mice [14].

Several studies also reveal that human pathogens can colonize plant tissues at wound sites and through natural openings during pre-/post-harvest processing, packaging, and transportation [25–29]. Once they have colonized in the plant tissue, many microorganisms could not be completely removed by washing [30]. Thus, fresh fruits or vegetables could be an important source of fungal human pathogens. Human pathogens on plants (HPOPs) have the ability and possibility to cohere, penetrate, colonize, and infect plants [27], which could be a risk factor for farmers and consumers [4,31–34]. Contamination of fruit and vegetables by human pathogenic bacteria including: *Escherichia coli*, *Clostridium* spp., *Listeria monocytogenes*, *Salmonella* spp, *Shigella* spp, *Staphylococcus aureus*, or *Yersinia* spp. has become more common [35]. To date, information regarding how or whether or not FFHP colonize vegetables remains limited.

The objectives of this study were: (1) to develop a molecular marker that can be selectively distinguish human opportunistic *F*. *oxysporum* (HOFo) isolates from other strains of *F*. *oxysporum*; (2) to assess the interaction and colonization of tomato and cucumber tissues by a HOFo isolate; and (3) to examine the sensitivity of HOFo isolates to two agro-fungicides.

## Materials and methods

### Sources of Taiwan human opportunistic *Fusarium oxysporum* (HOFo) isolates

Three Taiwan HOFo isolates: MCC2074, CGMHD0282 and CGMHD0413, isolated from blood, skin, and trachea, respectively, were provided by Chang Gung Memorial Hospital, Taoyuan, Taiwan. Six international HOFo isolates from three areas: NRRL25749 (Belgium), NRRL26361 (USA), NRRL26362 (USA), NRRL26376 (USA), NRRL26386 (USA), and NRRL26551 (Canada) were obtained from Agricultural Research Service Culture Collection, United States Department of Agriculture (Table 1). All fungal isolates were single-spore isolated and propagated on potato dextrose agar (PDA, Difco, USA) at 24 to 28°C with 12 hr light/dark. Conidia for inoculum were washed from the 10-14-day-old plate cultures and suspended in sterile distilled water. Mycelia for DNA extraction were taken from the agar surface of a 10-14-day-old culture of each isolate grown on PDA slant.

**Table 1:** Name, formae speciales, isolation source, pathogenicity and PCR reaction with Primer 5/ST33-T of *Fusarium oxysporum*, *Fusarium* spp. and other fungi used in this study.

Tested fungi	Isolate ^1^	Source / Pathogenicity ^2^	PCR reaction ^3^
*F*. *oxysporum* f. sp. *anoectochili*	St61, St62, St63, St92, Le91, Le92	Jewel orchid / +	−
*F*. *oxysporum* f. sp. *chrysanthemi*	Foch11-28	Garland chrysanthemum / +	−
*F*. *oxysporum* f. sp. *conglutinans*	Focn15, Focn20	Radish / +	−
*F*. *oxysporum* f. sp. *cubense*	Focb-25	Banana / +	−
*F*. *oxysporum* f. sp. *cucumerinum*	ATCC204376, ATCC204377, ATCC204378, Foc0812	Cucumber / +	−
*F*. *oxysporum* f. sp. *gladioli*	Fog051, Fog053	Gladiolus / +	−
*F*. *oxysporum* f. sp. *lactucae*	Fola103-7	Lettuce / +	−
*F*. *oxysporum* f. sp. *lilii*	FoliG16	Lily / +	−
*F*. *oxysporum* f. sp. *luffae*	Folu227, Folu308, Folu638, Folu572, Folu575, Folu601, Folu608, Folu701, Folu702, Folu1601	Loffah / +	−
*F*. *oxysporum* f. sp. *lycopersici*	Foly146	Tomato / +	−
*F*. *oxysporum* f. sp. *melonis*	Fom3, Fom6	Muskmelon / +	−
*F*. *oxysporum* f. sp. *momordicae*	Fomo34	Bitter gourd / +	−
*F*. *oxysporum* f. sp. *niveum*	FonH0103	Water melon / +	−
*F*. *oxysporum* f. sp. *phaseoli*	Fop04, Fop06, Fop07	Snap bean / +	−
*F*. *oxysporum* f. sp. *radices-cucumerinum*	ATCC204371, ATCC204372	Cucumber / +	−
*F*. *oxysporum* f. sp. *tracheiphilum*	F74, F75, F94, F96, F97, F98, F99, F100, F101, F102, F103, F104, F105	Asparagus bean / +	−
Nonpathogenic *Fusarium oxysporum*	SPA7, Fo7, Fo95013, Fo95015, Fo95022, OSS11, OSS12, OSS14, AV 0131, Fo276, HS33	Plant tissues or rhizosphere soils / −	−
*Fusarium oxysporum*	NRRL25749, NRRL26361, NRRL26362, NRRL26376, NRRL26386, NRRL26551, MCC2074, CGMHD0282, CGMHD0413	Human / ND	+
*F*. *chlamydospores*	14023, 14038	Rice /−	−
*F*. *fujikuroi*	14099, 14146	Rice / +	−
*F*. *graminearum*	DAYA350-2	Wheat / +	−
*F*. *moniliforme*	STP01	Corn feed / ND	−
*F*. *solani*	Le54	Jewel orchid / +	−
*F*. *verticillioides*	939229–3	Cymbidium orchid / +	−
*Aspergillus aculeatus*	En-A	Soil / ND	−
*A*. *flavus*	13049, 14038	Rice / ND	−

^1^ Pathogenic strains of *Fusarium oxysporum* were isolated from soil, seed, or diseased host tissue. The other *F*. *oxysporum* strains were isolated from soil or healthy plant tissue.

^2^ *F*. *oxysporum* isolates were tested for their pathogenicity using the root dip assay on their respective hosts, “+” = positive for pathogenicity; “−” = no disease development; “ND” = not tested.

^3^ The “+” = PCR product of the expected size obtained; “−” = no PCR product of the expected size obtained.

### Extraction of total DNA from HOFo isolates and other fungi

Fungal DNA was extracted by the method of Wang et al. [36] with some modifications. Mycelium (0.1 g) was transferred into a 1.5 ml microcentrifuge tube containing 500 μl lysis buffer (200 mM Tris-HCl, 50 mM ethylenediaminetetraacetic acid, 200 mM NaCl, 1% n-lauroylsarcosine sodium salt at pH 8.0) and incubated at 65°C for 30 min. Phenol/chloroform/isoamyl alcohol (25:24:1) (500 μl) was added to each microtube and agitated. The mixture was centrifuged at 18,000 *g* for 10 min at 4°C (Rotor: Nr. 12154, Sigma 3k20). The supernatant was transferred into a new microtube with 0.6 fold of isopropanol and incubated overnight at -20°C. For DNA pellet collection, the mixture was centrifuged at 18,000 *g* for 5 min at 4°C and the supernatant removed. The DNA pellet was washed with 500 μl of -20°C 70% ethanol and centrifuged at 18,000 *g* for 1 min at 4°C. The supernatant was removed and the DNA pellet placed in a laminar flow hood for drying after which it was resuspended in 30 μl sterile water and stored at -20°C for analysis later.

### Primer design and PCR amplification of Taiwan HOFo isolates

A previous study indicated that HOFo isolates could be separated into 9 sequence types (STs), ST-7, -33, -62, -126, -128, -183, -295, -296, -297, based on TEF-1α and IGS region sequences [37]. Initial results showed that Taiwan HOFo type isolate MCC2074 matched the ST33 reference isolates in GenBank [http://www.ncbi.nlm.nih.gov/genbank/index.html] based on IGS region sequences. To develop molecular markers, the primers CNL12/CNS1 [38] were used to amplify the total IGS region sequences of three Taiwan HOFo isolates. In this study, 14 strains of ST33 HOFo IGS sequences were obtained from the NCBI GeneBank database and used to compare with the HOFo isolate MCC2074 (Accession number: MT254057)in Taiwan. Accession numbers of these references isolates are as follows: AY527704, AY527684, AY527660, AY527654, AY527628, AY527627, AY527624, JN235411, JN235417, JN235424, JN235432, JN235438, JN235462, and JN235493. For comparison, six different formae speciales of *F*. *oxysporum* (each one isolate of f. sp. *cubense* (FJ985560), *cucumerinum* (FJ985613), *lycopersici* (FJ985588), *melonis* (FJ985448), *momordicae* (FJ985498), and *raphani* (FJ985463)), and one nonpathogenic isolate of *F*. *oxysporum* with biological control activity in Taiwan was also included (KC622301). The sequences were compared and aligned with the above-mentioned references isolates and used to design new primers based on software Clustal X 1.81.

### Specificity and sensitivity of the primers for HOFo isolates

Specificity of primers, Primer 5/ST33-R was evaluated by testing on 71 *F*. *oxysporum* isolates, 8 *Fusarium* spp. isolates and 3 other fungi (Table 2). Among the 71 *F*. *oxysporum* isolates, there were 16 formae speciales including those that cause Fusarium wilt in Asteraceae (2 isolates), Brassicaceae (2 isolates), Cucurbitaceae (20 isolates), Leguminosae (16 isolates), lily (1 isolate), tomato (1 isolate), banana (1 isolate), gladiolus (2 isolates) and jewel orchid (6 isolates); 11 nonpathogenic *F*. *oxysporum* isolates; and 9 HOFo isolates. The PCR reaction volume was 25 μl, including100 ng DNA, PCR Master Mix II (1.25 μl of Taq DNA polymerase, reaction buffer, 1.75 mM MgCl_2_, 200 μM dNTP and enzyme stabilizer (Genemark Technology Co., Ltd, Taiwan)) and primers (0.2 μM). The PCR conditions were as follows: denature at 95°C 2 min; 95°C 30 sec; 63.7°C 1 min; 72°C 45 sec for 30 cycles and final extension at 72°C 10 min. All the PCR reactions were conducted three times to confirm reproducibility. For evaluation of the new primers sensitivity, the quality of MCC2074 DNA was quantified in GeneQuant 100 classic spectrophotometer (GE Healtcare), and diluted into several concentrations from 100 to 10^−3^ ng using 1 μl of each concentration in each treatment as template DNA in a 25 μl PCR reaction volume. The sensitivity text of new primers were replicated three times with independent dilutions.

**Table 2:** Distribution of HOFo isolate MCC2074 based on specific primers Primer 5/ST33-R in ‘Yu-Nyu’ cherry tomato plant tissues following hypocotyl inoculation.

	PCR amplification following inoculation^2^		
Tissue sample^1^	2wk	3wk	4wk
Hypocotyl 1	+	+	+
CN-1	+	+	+
CN	+	+	+/-
Stem 1	+/-	+	+/-
Stem 2	ND	+/-	+/-

^1^ Hypocotyls and stems of tomato seedlings were cut into 1 cm sections from the bottom to the top; “CN” = cotyledonary node, and cm = distance from CN

^2^ “+” = MCC2074 detected in all three replicate samples; “−” = no PCR product of MCC2074 was amplified in any of thethree replicate samples; and “+/−” = MCC2074 was detected in some but not all replicate samples.

### Colonization of Taiwan HOFo isolate MCC2074 in tomato plants

To assess the capability of Taiwan HOFo isolate MCC2074 to colonize tomato plants, they were inoculated by three methods as follows: (1) Hypocotyl inoculation, twenty 7-10-day-old cherry tomato seedlings (‘Yu-Nyu’, Known-You Seed Co. Ltd) were cut off just above the roots and the hypocotyls immersed in a 5x10^4^/ml conidial suspensions for 30 min, transplanted into sterile peat moss in 5 cm^3^ pots, covered with humidity domes to maintain high humidity, and placed in the greenhouse at 25–35°C. Domes were removed after 3 days and plants examined for disease symptoms and MC2074 colonization 7 days after transplanting. A parallel set of plants handled in the same way but inoculated with *F*. *oxysporum* f. sp. *lycopersici* (isolate of Fol146) and rated for disease severity as follows: 0 = health, 1 = cotyledon and first leaf with yellowing symptom, 2 = stunting or <1/2 leaves with the yellowing symptom, 3 = stem yellowing, vascular discoloration, and >1/2 leaves with wilt symptoms, and 4 = plant wilted and dead.; (2) Soil drench inoculation, seeds of ‘Yu-Nyu’ cherry tomato and ‘Farmers 301’ cooking tomato (Known You Seed, Kaohsiung, Taiwan) were sown into sterile peat moss in 5 cm^3^ pots and 25 plants of each variety inoculated by pipetting 3 ml of a 1x10^5^/ml conidial suspension into the growth medium at the base of each plant at the 2-leaf stage. Plants were examined for colonization 7 days after inoculation; (3) Infested soil inoculation, inoculum was prepared by infesting a sterile mixture of oat grain, sand, and water (100:100:20, w/w/v) with a conidial suspension (10 ml with 1x10^6^ spores/ml) and incubated for 2 weeks (shaken briefly each week) in the greenhouse at 25–28°C. The inoculum was combined in equal quantities with sterile soil and incubated another 2 weeks in the same environment. Propagule density was estimated by suspending 1 g of the infested soil in 10 ml sterile water and spreading 1 ml aliquots of the suspension onto quintozene (PCNB) peptone agar plates. Infested soil was mixed with sterile soil to achieve 10^4^ propagules per gram. Twenty-five ‘Yu-Nyu’ tomato plants were transplanted at the 2-leaf stage into the infested soil and examined for colonization after 2 wk in the greenhouse.

### Inoculation of tomato and cucumber fruit by Taiwan HOFo isolate MCC2074, *F*. *oxysporum* f. sp. *lycopersicum* and *F*. *oxysporum* f. sp. *cucumerinum*

Fruit of ‘Yu-Nyu’ cherry tomato and ‘Swallow’ cucumber (Known-You Seed Co. Ltd.) were inoculated with Taiwan HOFo isolate MCC2074 to assess its ability to colonize the fruit. Prior to inoculation, tomato and cucumber fruit were soaked in 1% NaClO solution for 30 min then washed with sterilized water. Surfaces of fruit were abraded with carborundum by a cotton swab to mimic injuries that may occur during harvest and postharvest handling. The injured fruits were then immersed in a 1x10^6^ /ml conidial suspension in water with 0.1% tween 20 for 30 sec. Inoculated fruits were dried in a laminar flow hood then incubated in a moist chamber at 28°C without light for 5 days before interior fruit structures were assessed for fungal colonization. An additional set of tomato and cucumber fruit handled in the same way were inoculated with *F*. *oxysporum* f. sp. *lycopersicum* isolate Foly146 and *F*. *oxysporum* f. sp. *cucumerinum* isolate Foc0812, respectively.

### Assays of tomato and cucumber tissues for fungal colonization by specific primers

Primer 5/ST33-R, specific for amplification of HOFo isolates, was used to assay various plant parts for colonization following inoculation. Tomato and cucumber fruit inoculated with Foly146 and Foc0812 were assayed for colonization using primer FIGS11/12 [36]. Parts of inoculated tomato plants assayed included root, hypocotyl, cotyledon, and stem. One-cm-long tissue samples of each part were collected and sterilized by immersing in a mixture 95% ethanol and 5.25% NaClO (1:1 v/v) for 15 sec prior to DNA extraction. Interior tomato and cucumber fruit tissues assayed for colonization included 1 g samples from pericarp wall, locular cavity, and columella. The DNA extraction was by DNA extraction kit (Quick-DNA Miniprep Kit, ZYMO, USA). The PCR reaction volume was 25 μl and included PCR Master Mix II (Genemark Technology Co., Ltd, Taiwan), DNA and 0.2 μM primers. The PCR reacting condition followed 95°C 2 min; 95°C 30 sec, 63.7°C 1 min, 72°C 45 sec, 35 cycles; 72°C 10 min (PX2 Thermal cycle, Thermo Electron Corporation). Colonization sites were associated with the specific DNA band showing after PCR amplification.

### Sensitivity of Taiwan HOFo isolates to agro-fungicides

In clinical medicine, azole fungicides such as fluconazole, itraconazole, voriconazole etc. are often used to treat human fungal diseases [11,39]. Azoles are also important for control of plant fungal diseases in agriculture environment (FRAC Code List ©*2019). We examined sensitivities of Taiwan HOFo isolates to two agro-fungicides, azoxystrobin (C3, FRAC code 11) and difenoconazole (G1, FRAC code 3). Sensitivities of these isolates were compared to those of international HOFo isolates from NRRL and *F*. *oxysporum* f. sp. *tracheiphilum* isolate Fot60 (Huang, personal communication). Three mm diameter agar discs from 7-day-old Taiwan HOFo cultures grown on PDA at 28°C with 12 h day/night were transferred twice onto three replicate plates of PDA amended with 1, 10, 100 and 500 mg a.i./L of each fungicide and incubated at 28°C without light. Inhibition of fungal growth was based on colony diameters after a 3-day incubation. The EC_50_ for each fungicide was calculated using log-linear model software kindly supplied by Dr. Hsin Chi (Taiwan)

## Results

### Identification and sensitivity of HOFo isolates by specific primers

**Fig 1:**
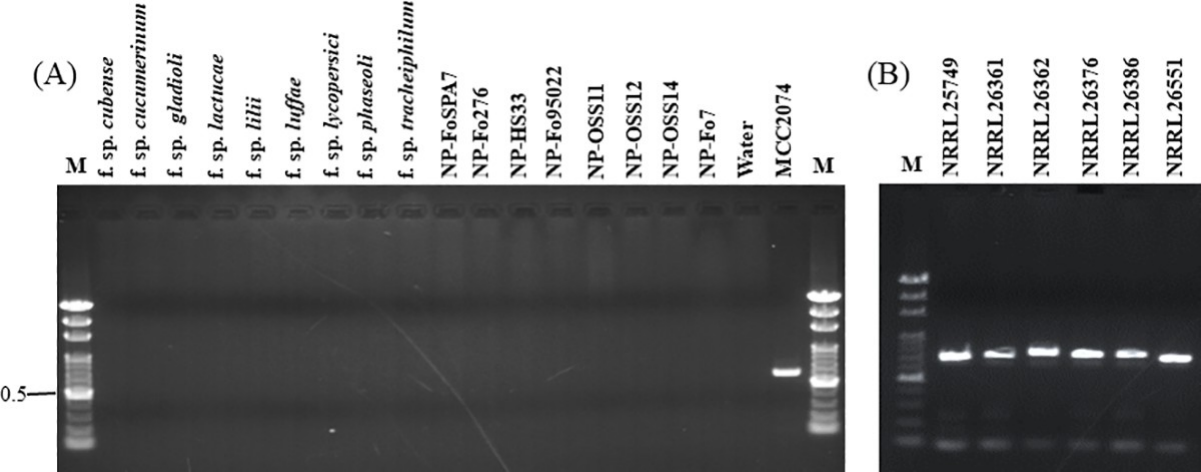
Specificity of the new designed primer pair. Agarose gels showing the amplification products from Polymerase Chain Reaction (PCR) using genomic DNA from isolates of 9 *formae speciales*, including *cubense*, *cucumerinum*, *gladioli*, *lactucae*, *lilii*, *luffae*, *lycopersici*, *phaseoli* and *tracheiphilum*, eight nonpathogenic strains of *Fusarium oxysporum* (*Fo*), and one Taiwan HOFo isolate MCC2074. (A)642 bp DNA product of Taiwan HOFo isolate MCC2074 amplified by the new primer pair Primer 5 and ST33-R. (B) Six reference isolates of HOFo from NRRL collection center could be detect and amplify a specific PCR product by Primer5/ ST33-R specific primers. The numbers on the left are the molecular weights (Kb) of the Gen-100 bp DNA ladder (GeneMark) (lane M).

**Fig 2:**
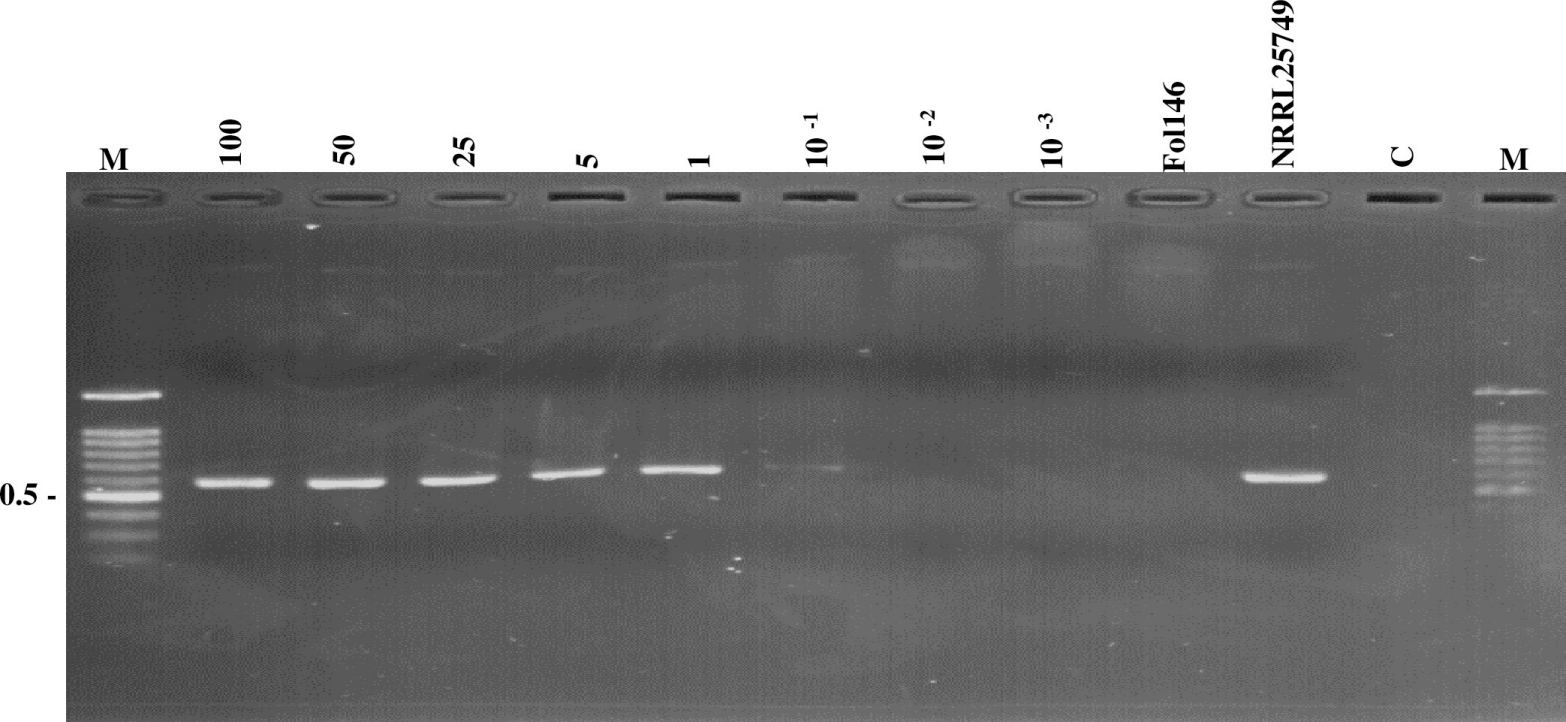
Sensitivity of the newly designed primers. Agarose gel showing the sensitivity of polymerase chain reaction (PCR) using the genomic DNA of a human opportunistic *Fusarium oxysporum* (HOFo) and the primer pair Primer 5/ST33-R: Amplification of a decreasing amount of the HOFo isolate MCC2074 DNA ranging from 100 to 10^−3^ ng. The numbers on the left correspond to the molecular weight (kb) of the Gen-100 ladder (lane M). Lanes Foly146 and NRRL25749, the amplification controls for the plant pathogenic isolate of F. *oxysporum* f. sp. *lycopersici* and HOFo DNA, respectively. Lane C, control reaction with no template DNA.

Fourteen strains of HOFo intergenic spacer (IGS) region sequences obtained from NCBI GeneBank database were compared with the HOFo isolated from Taiwan and other *Fo* formae special isolates. Comparison of IGS nucleotide variation with other plant pathogenic and nonpathogenic *Fo* isolates using Clustal X 1.81 alignment software, two primers, designated Primer 5 (5' -GAGATATGTGGTTCAGGGTAGG- 3') and ST33-R (5' -TTTCAGCTACCCTAGAGTGATG—3'), were designed to identify and detect HOFo isolates (S1 Fig). The primer pair Primer 5/ST33-R was found to amplify a 642 bp DNA fragment from three HOFo isolates originally cultured from Taiwan and six international HOFo reference isolates (Fig 1 and Table 1). No products were obtained from DNA prepared other 16 formae speciales and 11 nonpathogenic isolates of *F*. *oxysporum* using Primer 5 and ST33-R. The primer pair Primer 5/ST33-R failed to amplify any DNA product from genomic DNA prepared from 51 different formae speciales and 11 nonpathogenic isolates of *F*. *oxysporum*, 9 HOFo isolates, 8 isolates of other *Fusarium* spp. and 3 isolates of *Aspergillus* spp. (Table 1). The PCR sensitivity result showed that the Primer 5 and ST33-R could amplify the 642 bp fragment from as little as 100 pg (10^−1^ ng) template DNA in a 25 μl reaction mixture (Fig 2).

### Virulence assessment of Taiwan HOFo isolate MCC2074 and *F*. *oxysporum* f. sp. *lycopersici* on tomato

Fungal virulence was assessed by hypocotyl inoculation. Hypocotyls of cherry tomato plants cv. ‘Yu-Nyu’ cherry tomato plants were cut off and inoculated by immersion in conidial suspensions prepared from *F*. *oxysporum* f. sp. *lycopersici*isolate Foly146 or the HOFo isolate MCC2074. Tomato plants inoculated with Foly146 developed typical fusarium wilt symptoms, including yellowing, wilting, and plant death (S2 Fig) with disease severity ratings of 50% at 2 weeks and 73% at 3 and 4 weeks after inoculation. By contrast, tomato plants inoculated with MCC2074 did not express any visible symptoms. PCR analysis with the Primer 5/ST33-R primer pair revealed that an expected 642 bp DNA fragment, indicative of the presence of MCC2074, could amplified from DNA samples prepared from hypocotyl, cotyledonary node, and stem tissues of a tomato plant after being inoculated with MCC2074 (Table 2).

Fungal virulence was also assessed by drench inoculation. Tomato seedlings cv. ‘Yu-Nyu’ (cherry tomato) and cv. ‘Farmers 301’ (cooking tomato) at the 2-leaf stage were inoculated by soil drenching with conidial suspensions prepared from MCC2074. No visible symptoms were observed. The 642 bp DNA fragment could be amplified from roots and lower hypocotyl tissues of both cultivars with the Primer 5/ST33-R primer pair 1, 2, 3, and 4 weeks after inoculation (Table 3). Amplification of the 642 bp DNA fragment from samples prepared from cotyledonary node and hypocotyls tissue below the node was inconsistent over the 4-week period. The 642 bp DNA fragment was amplified from stem tissues collected from ‘Yu-Nyu’ cherry tomato samples 3 and 4 weeks after inoculation. Fungal virulence was assessed by planting tomato seedlings in soils infested with MCC2074. The results revealed that the 642 bp DNA fragment was amplified from the roots of ‘Yu-Nyu’ cherry tomato plants 4, 8, and 12 weeks and from hypocotyl and cotyledonary node tissue 12 weeks after transplant (Table 4).

**Table 3:** Distribution of HOFo isolate MCC2074 in tomato plant tissues based on specific primers Primer 5/ST33-R following soil drench inoculation.

Plant tissues^a^	PCR amplification following inoculation^b^							
	Cherry tomato (Yu-Nyu)				Cooking tomato (Farmers 301)			
	1wk	2wk	3wk	4wk	1wk	2wk	3wk	4wk
Root	+/-	+/-	+	+/-	+/-	+/-	+/-	+/-
Hypocotyl 1	+/-	+/-	+	+/-	+/-	+/-	+	+/-
CN-1	+/-	-	-	+/-	+/-	-	+/-	-
CN	+/-	-	+/-	-	+/-	-	+/-	-
Stem 1	-	-	+/-	+/-	-	-	-	-

^a^ The hypocotyl and stem tissues were cutted into pieces (about 1 cm each) from the bottom to the top. The numbers indicated the height of the tissues. The “CN” means the cotyledonary node, and the “CN-1” means the region that under 1 cm of the CN.

^b^ The primers Primer5/ST33-R could amplified 642 bp DNA product ofMCC2074. The symbol “+” means that MCC2074 could be detected from this part tissues of all three replicate samples, the symbol “−” means that no PCR product of MCC2074 were amplified of all three replicate samples, and the symbol “+/−”means that MCC2074 could be detected from this part tissues of some replicate samples.

**Table 4:** Distribution of HOFo isolate MCC2074 in tomato plant tissues based on specific primers Primer 5/ST33-R following soil infestation inoculation.

Plant tissues^a^	PCR amplificationfollowing inoculation^b^		
	4 wk	8 wk	12 wk
Root	+	+	+
Peg	+/-	+/-	-
CN-1	-	-	+/-
CN	-	-	+/-
Stem 1	-	-	-
Stem 30	-	-	-
Stem 60	-	-	-
Stem 90	ND	-	-
Fruit	ND	-	-

^a^ The hypocotyl and stem tissues were cutted into pieces (about 1 cm each) from the bottom to the top. The numbers indicated the height of the tissues. The “CN” means the cotyledonary node, the “CN-1” means the region that under 1 cm of the CN, and the “Stem 30” means the region that above 30 cm of the CN.

^b^ The primers Primer5/ST33-R could amplified 642 bp DNA product ofMCC2074. The symbol “+” means that MCC2074 could be detected from this part tissues of all three replicate samples, the symbol “−” means that no PCR product of MCC2074 were amplified of all three replicate samples, and the symbol “+/−”means that MCC2074 could be detected from this part tissues of some replicate samples.

### Colonization of tomato and cucumber fruit by HOFo isolate MCC2074, *F*. *oxysporum* f.sp. *lycopersici* (Fol) and *F*. *oxysporum* f.sp *cucumerinum* (Foc)

Tomato and cucumber fruits were inoculated with conidial suspensions prepared from MCC2074, Fol, or Foc. PCR with the 5/ST33-R primer pair amplified the 642-bp DNA fragment from pericarp, locular cavity, and columella of tomato fruit and pericarp and locular cavity of cucumber fruit 5 days after inoculation (Table 5). Fol and Foc were detected solely from pericarp and locular cavity tissues of tomato and cucumber, respectively. No fungus was detected in seed samples taken from inoculated tomato or cucumber.

**Table 5:** Colonization of tomato and cucumber fruit by HOFo isolate MCC2074, *F*. *oxysporum* f. sp. *lycopersici* and f. sp. *cucumerinum* following surface inoculation^1^.

Treatment	PCR reaction 5 days after inoculation^2^							
	cucumber				tomato			
	P^3^	L	C	S	P	L	C	S
Water	**-**	**-**	**-**	**-**	**-**	**-**	**-**	**-**
Foc0812	+	+	**-**	**-**	+	**-**	**-**	**-**
Foly146	+	**-**	**-**		+	+	**-**	**-**
MCC2074	+/**-**	+/**-**	**-**	**-**	+/**-**	+/**-**	+/**-**	**-**

^1^ Cumber and tomato fruits were laid paper disks (0.2 cm) with 50 *μl* fungal spore suspension (1x10^6^ spores/ml)

^2^The primers FIGS11/12 amplified a 700 bp DNA product of Foc0812 and Foly146, and the Primer 5/ ST33-R could amplified 650 bp DNA product of MCC2074. The symbol “+” means that target isolate could be detected from this part tissues of all three replicate samples, the symbol “−” means that no PCR product of target isolate were amplified of all three replicate samples, and the symbol “+/−”means that target isolate could be detected from this part tissues of some replicate samples.

^3^“P” = Pericarp wall; “L” = Locular cavity; “C” = Columella; “S” = Seeds

### Sensitivity of HOFo isolates to agro-fungicides

Fungicide sensitivity assays revealed that HOFo isolates were more sensitive to difenconizol than to azoxystrobin than other test Fusarium strains (Table 6). Mycelial growth of international isolates from NRRL was completely inhibited at 10 mg a.i./L of difenconizol whereas 100 ppm was required to completely inhibit the growth of Taiwan HOFo isolates. All test fungal strains grew poorly on medium amended with 1, 10, 100, or 500 mg a.i./L of azoxystrobin. However, it appeared that Taiwan HOFo isolates were less sensitive to azoxystrobin compared to the international isolates. Taiwan isolate Foc0812 and Fot60, isolated from the conventional farming area with cucumber and asparagus bean production farms in Nantou and Pingtung county, respectively, displayed sensitivity to both test fungicides at levels similar to the international isolates.

**Table 6:** Sensitivity of mycelial growth of HOFo isolates to azoxystrobin and difenoconazole.

Location	Isolate	EC_50_ (mg a.i./L)^1^	
		Azoxystrobin	Difenoconazole
Belgium	NRRL25749	2.0	<1
USA	NRRL 26361	11.0	<1
USA	NRRL 26362	29.4	<1
USA	NRRL 26376	36.5	<1
USA	NRRL 26386	<1	<1
Canada	NRRL 26551	16.3	<1
Taiwan	MCC2074	>500	<1
Taiwan	CGMHD0282	>500	-^2^ (<10)
Taiwan	CGMHD0413	>500	- (<10)
Taiwan	Foc0812	4.0	<1
Taiwan	Fot60	1.4	<1

^1^EC_50_: Effective concentration of the tested fungicide at which inhibition occurs at 50% mycelial growth of HOFo.

^2^“−”: Non determination.

## Discussion

The sequence types show that the three Taiwan HOFo isolates belong to the sequence type ST33. Short et al. [37] reported that ST33 is the predominant clinical type of *F*. *oxysporum* species complex (FOSC). Although only three isolates were examined, we suspect that clinical ST33 is the predominant type in Taiwan. In this study, the primer pair (Primer 5/ST33-R) has been shown to be highly specific to the ST33 isolates. Other studies have demonstrated that sequence types could be differentiated by TEF-1α and IGS regions [37,40]. The two regions displaying high levels of nucleotide diversity are suitable for fungal identification, classification and specific primer design [36,41]. Here, the entire IGS region of the HOFo isolates was sequenced and compared with IGSs of other *F*. *oxysporum*. Consequently, two primers Primer 5 and ST33-R were designed to detect the HOFo isolates from Taiwan and ATCC isolates. The primers show higher degrees of specificity because they are unable to amplify any products from genomic DNA prepared from other formae speciales or nonpathogenic *F*. *oxysporum*.

Tomato plants inoculated with the clinical HOFo isolate MCC2074 show no visible disease symptoms although the fungal isolate is able to colonize tissues in the lower part of tomato plants. Of three inoculation methods tested, hypocotyl inoculation after the roots are cut off results in the most rapid and extensive colonization of the fungus in the lower stem sections above the cotyledonary node (Table 2). Although the method of inoculation affects the rate and the extent of tissue colonization, the overall data show that colonization by the isolate MCC2074 is restricted to the lower part of tomato plants. This suggests that MCC2074 fails to move systemically through the vascular system as *F*. *oxysporum* f. sp. *lycopersici* does. Previous studies have shown that proteins secreted-in-xylem (SIX) are closely related to pathogenicity of *F*. *oxysporum* f. sp. *lycopersici* [42,43]. The primer pair SIX6-F/SIX6-R [44] fails to amplify the SIX6-coding gene (Chung, unpublished data) in any of the HOFo isolates used in this study, suggesting that the clinical HOFo isolates likely do not have the ability to cause diseases in plants. Nonpathogenic *F*. *oxysporum* has been shown to colonize crop tissues because of the lack of the SIX6-coding gene [36,45]. *Fusarium* spp. could be endophytic fungi commonly found in many different plants [46,47], and it has been noted that, if inoculum is available, there is a risk for human pathogens or human opportunistic pathogens to contaminate plant products or enter into plant tissues [48,49].

In this study, the tomato and cucumber fruit with small surface wounds were extensively colonized following surface inoculation with HOFo isolate MCC2074. Although interior tissues of the fruit were colonized, they exhibited no disease symptoms. These results show that there is a potential for symptomless tomato and cucumber fruit intended for human consumption to be infected with HOFo isolates. Our study also indicated that the tested HOFo isolates could survive in soils with chlamydospores. Previous studies have demonstrated that the human bacterial [48,49,50] or fungal [51,52] pathogens could adhesive on the surface of plants by the contamination of animal excrement, plant debris, and soils. Although the HOFo MCC2074 isolate could not cause symptoms in plants or fruit, the MCC2074 has the ability to overcome the physical and chemical barriers of unsusceptible plants. The HOFo isolates could directly penetrate or through natural openings into plant or fruit tissue [1,27]. Springer et al. [51] and Xue et al. [52] reported that the human pathogen *Cryptococcus* could colonize inside of the living plant tissues, such as leaves and vascular, and the characteristics play an important role for *Cryptococcus* to complete sexual cycles and produce infectious propagules. If HOFo could persist in plant tissue, they might have chances to encounter with pathogenic *F*. *oxysporum* or nonpathogenic *F*. *oxysporum*, raising a concern about gene flow or gene exchanging [53]. Thus, plants serve as a platform where HOFo may contact with other *F*. *oxysporum* strains. In addition, the possibility of HOFo isolates to colonize in plant or fruit tissue may increase the risk for immunosuppressed patients who come to contact or eat the contaminated fruits and plants.

The fungicidal sensitivity of HOFo isolates to difenoconazole and azoxystrobin indicated that HOFo isolates were more tolerant to azoxystrobin than difenoconazole. This is interesting as azoxystrobin is an agricultural fungicide belonging to aryloxypyrimidine, which has a 4,6-diphenoxypyrimidine skeleton. Azoxystrobin impacts mitochondrial respiration by interrupting electron transfer between cytochromes b and c1 [54]. Difenoconazole is also an agricultural fungicide belonging to triazoles, which are demethylation inhibitor (FRAC Code List ©*2019). Triazoles are commonly used to treat human fungal diseases [11,39]. Resistance to azole drugs has been reported in certain human fungal pathogens [10]. Thus, the drug resistance of HOFo isolates to difenoconazole should be higher than azoxystrobin. However, the results show an opposite reaction between difenoconazole and azoxystrobin. This result indicates that the azoxystrobin-resistance might be inherent in HOFo isolates, and this phenomenon has been observed in *F*. *graminearum* [causing Fusarium head blight] with natural resistance to trifloxystrobin in Europe [55]. Thus, the HOFo isolates show a lower sensitivity to fungicides might also be associated with natural resistance. Previous studies have revealed that the strobilurin-resistance is associated with the point mutation at codons 129 and 143 in the *cyt* b gene [56–58] and alternative oxidase (AOX) [59–61]. The resistant mechanism warrant more research in future. In this study, the sensitivities of the Taiwanese HOFo isolates to azoxystrobin and difenoconazole at 1 and 10 mg a.i./L are lower than the USA HOFo isolates. These results demonstrate that the drug reaction of the HOFo isolates might dependent on the geographic conditions. The Taiwanese HOFo isolates might have the chance to expose to triazoles with a higher concentration over a long period of time than the USA isolates. It is necessary to collect more Taiwanese isolates to confirm the sensitivity to difenoconazole and other triazole fungicides.

## Supporting information

S1 Fig Nucleotide variation of human opportunistic *Fusarium oxysporum* (HOFo) isolates (MCC2074) and other reference Fo isolates. Nucleotide sequence alignment of the rDNA repeats encoding a part of the intergenic spacer region (IGS) for isolates of HOFo and plant pathogenic and nonpathogenic F. oxysporum (No. 2–15 represented HOFo isolates ST-33 group; No. 16 represented nonpathogenic F. oxysporum; No. 18–22 represented cucumerinum, lycopersici, cubense, melonis, momordicae and raphani, respectively. Lowercase letters indicate the nucleotide bases that differ between the HOFo, nonpathogenic and pathogenic Fo isolates. The dashes indicate base gaps. The dashed line region represented the sequence of specific primers, Primer5 (A) and ST33-R (B).
(TIF)

S2 Fig The Fusarium wilt symptoms on tomato. The 7-10-day-old cherry tomato seedlings (‘Yu-Nyu’, Known-You Seed Co. Ltd) were used hypocotyl cutting inoculation method to inoculate with F. oxysporum f. sp. lycopersici (isolate of Fol146) (5x10^4^/ml conidial suspensions) for 30 min. The diseased plants usually showed cotyledon and first leaf with yellowing, stunting, and wilting symptoms.
(TIF)

S1 Raw images (TIF)

S2 Raw images (TIF)

S3 Raw images (TIF)
