# Correction: Colonization of human opportunistic *Fusarium oxysporum* (HOFo) isolates in tomato and cucumber tissues assessed by a specific molecular marker

**DOI:** 10.1371/journal.pone.0244388

**Published:** 2020-12-16

**Authors:** Chao-Jen Wang, Chinnapan Thanarut, Pei-Lun Sun, Wen-Hsin Chung

The affiliation for the third author is incorrect. The correct affiliation is not indicated. Pei-Lun Sun is not affiliated with #3 but with: Department of Dermatology, Chang Gung Memorial Hospital, Linkou Branch and College of Medicine, Chang Gung Univerisity, Taoyuan, Taiwan.
