# Supplementary figures and images for: Colonization of human opportunistic Fusarium oxysporum (HOFo) isolates in tomato and cucumber tissues assessed by a specific molecular marker
Source: PLoS One. 2020 Jun 12;15(6):e0234517. doi: 10.1371/journal.pone.0234517 (PMC7292389; doi:10.1371/journal.pone.0234517)

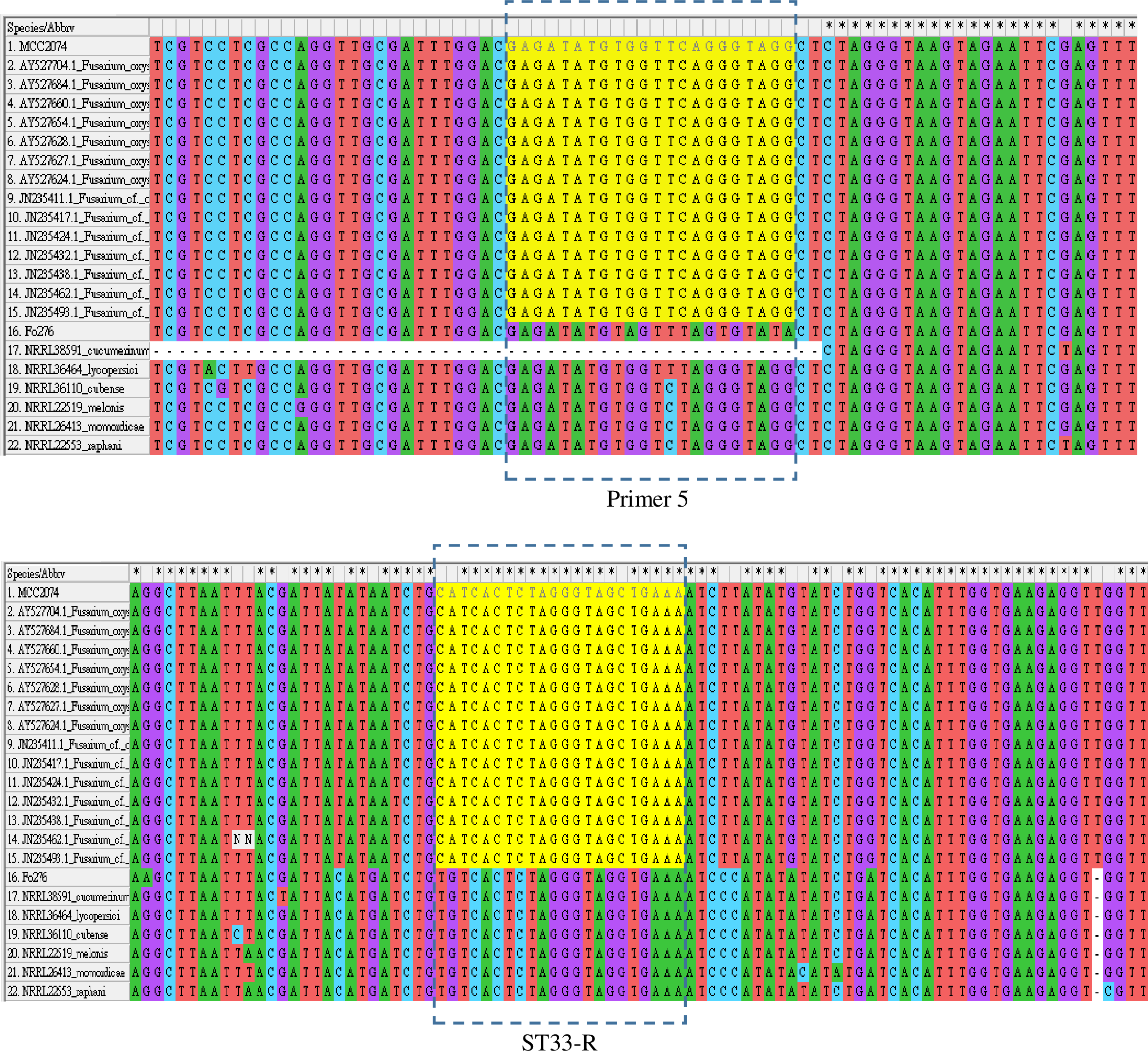

Supplement: S1 Fig — Nucleotide sequence alignment of the rDNA repeats encoding a part of the intergenic spacer region (IGS) for isolates of HOFo and plant pathogenic and nonpathogenic F. oxysporum (No. 2–15 represented HOFo isolates ST-33 group; No. 16 represented nonpathogenic F. oxysporum; No. 18–22 represented cucumerinum, lycopersici, cubense, melonis, momordicae and raphani, respectively. Lowercase letters indicate the nucleotide bases that differ between the HOFo, nonpathogenic and pathogenic Fo isolates. The dashes indicate base gaps. The dashed line region represented the sequence of specific primers, Primer5 (A) and ST33-R (B). (TIF) [file pone.0234517.s001.tif]

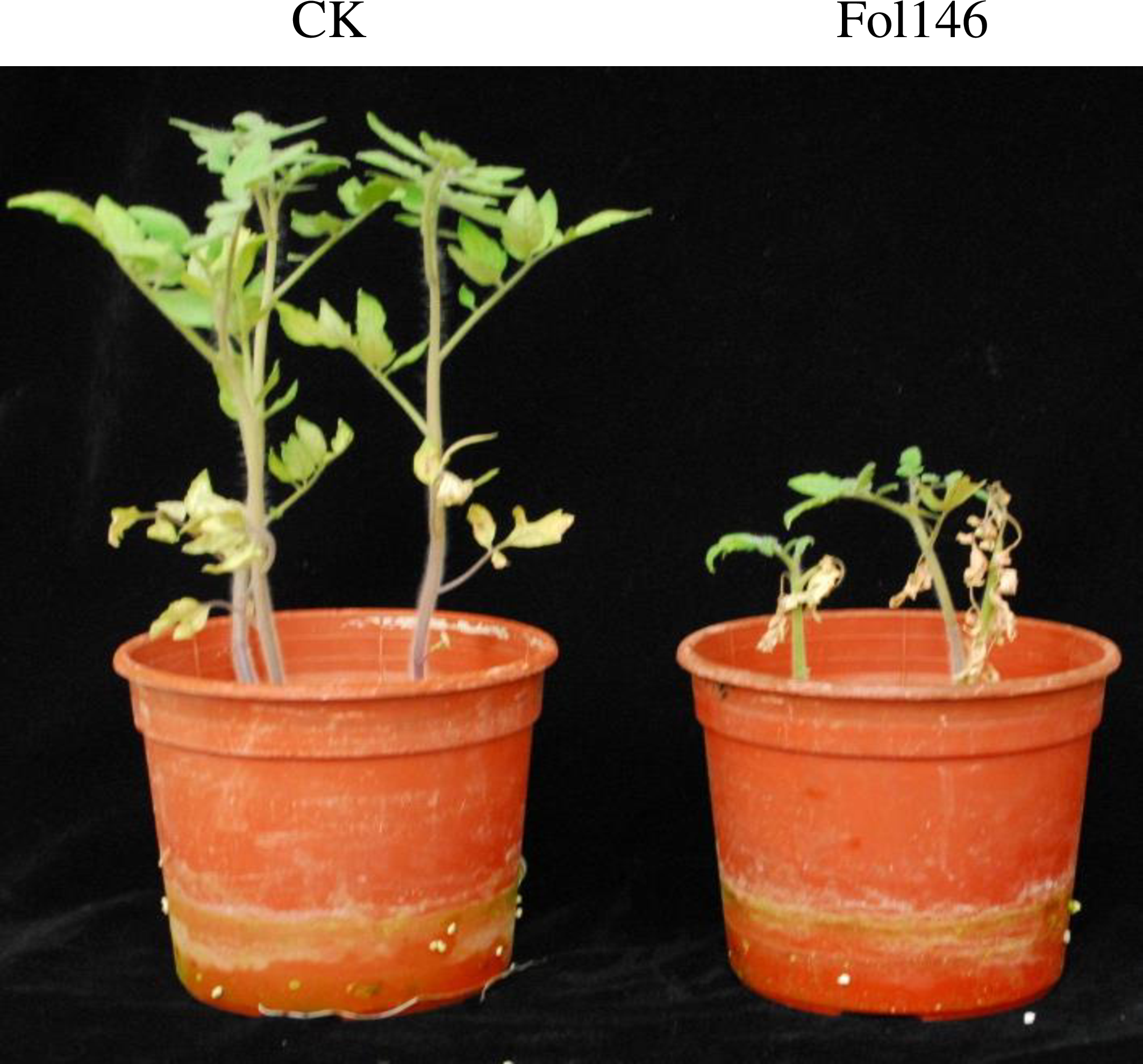

Supplement: S2 Fig — The 7-10-day-old cherry tomato seedlings (‘Yu-Nyu’, Known-You Seed Co. Ltd) were used hypocotyl cutting inoculation method to inoculate with F. oxysporum f. sp. lycopersici (isolate of Fol146) (5x10^4^/ml conidial suspensions) for 30 min. The diseased plants usually showed cotyledon and first leaf with yellowing, stunting, and wilting symptoms. (TIF) [file pone.0234517.s002.tif]

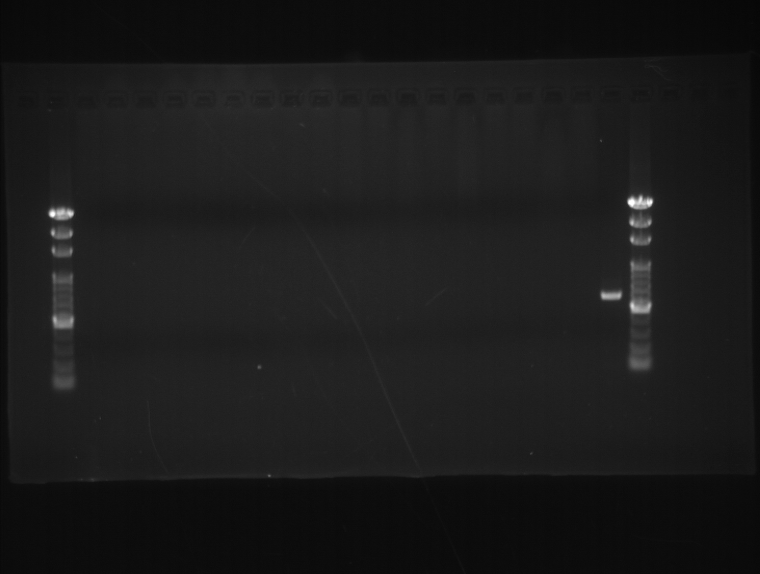

Supplement: S1 Raw images — (TIF) [file pone.0234517.s003.tif]

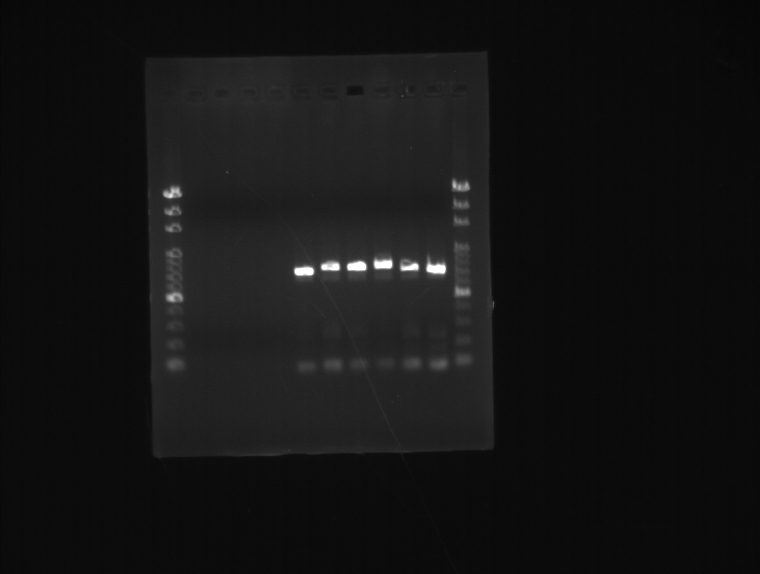

Supplement: S2 Raw images — (TIF) [file pone.0234517.s004.tif]

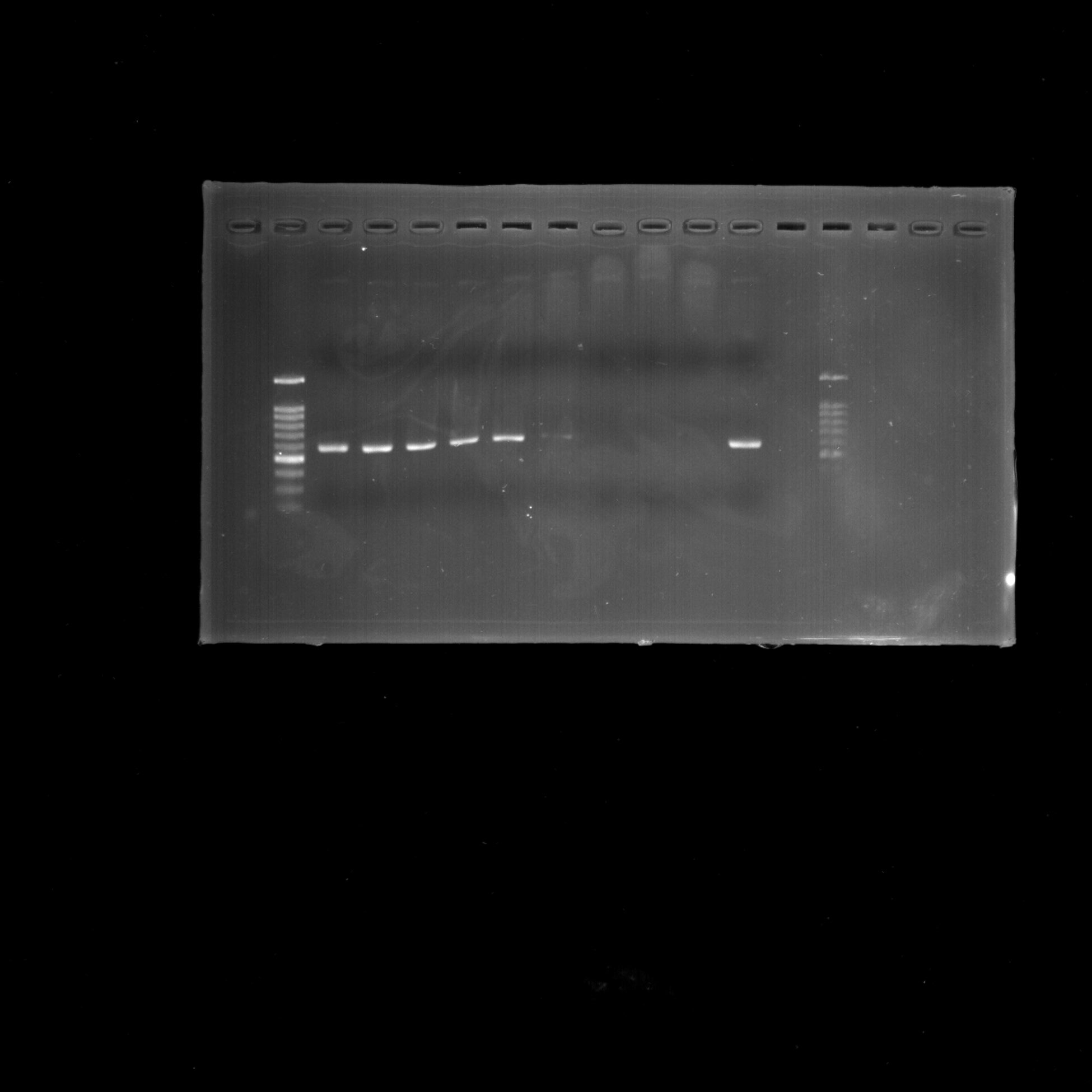

Supplement: S3 Raw images — (TIF) [file pone.0234517.s005.tif]
